# Identification of Ion Channel-Related Genes and miRNA-mRNA Networks in Mesial Temporal Lobe Epilepsy

**DOI:** 10.3389/fgene.2022.853529

**Published:** 2022-03-29

**Authors:** Zhengwei Su, Yinchao Li, Shuda Chen, Xianyue Liu, Ke Zhao, Ying Peng, Liemin Zhou

**Affiliations:** ^1^ Department of Neurology, The Seven Affiliated Hospital, Sun Yat-sen University, Shenzhen, China; ^2^ Department of Neurology, Sun Yat-sen Memorial Hospital, Sun Yat-sen University, Guangzhou, China

**Keywords:** temporal lobe epilepsy, ion channel, microRNA, MiR-27a-3p, bioinformatics

## Abstract

**Objective:** It aimed to construct the miRNA-mRNA regulatory network related to ion channel genes in mesial temporal lobe epilepsy (mTLE), and further identify the vital node in the network.

**Methods:** Firstly, we identified ion channel-related differentially expressed genes (DEGs) in mTLE using the IUPHAR/BPS Guide to Pharmacology (GTP) database, neXtProt database, GeneCards database, and the high-throughput sequencing dataset. Then the STRING online database was used to construct a protein-protein interaction (PPI) network of DEGs, and the hub module in the PPI network was identified using the cytoHubba plug-in of Cytoscape software. In addition, the Single Cell Portal database was used to distinguish genes expression in different cell types. Based on the TarBase database, EpimiRBase database and the high-throughput sequencing dataset GSE99455, miRNA-mRNA regulatory network was constructed from selected miRNAs and their corresponding target genes from the identified DEGs. Finally, the rats were selected to construct chronic li-pilocarpine epilepsy model for the next stage experimental verification, and the miR-27a-3p mimic was used to regulate the miRNA expression level in PC12 cells. The relative expression of miR-27a-3p and its targeting mRNAs were determined by RT-qPCR.

**Results:** 80 mTLE ion channel-related DEGs had been screened. The functional enrichment analysis results of these genes were highly enriched in voltage-gated channel activation and ion transport across membranes. In addition, the hub module, consisting of the Top20 genes in the protein-protein interaction (PPI) network, was identified, which was mainly enriched in excitatory neurons in the CA3 region of the hippocampus. Besides, 14 miRNAs targeting hub module genes were screened, especially the miR-27a-3p deserving particular attention. miR-27a-3p was capable of regulating multiple mTLE ion channel-related DEGs. Moreover, in Li–pilocarpine-induced epilepsy models, the expression level of miR-27a-3p was increased and the mRNAs expression level of *KCNB1*, *SCN1B* and *KCNQ2* was decreased significantly. The mRNAs expression level of *KCNB1* and *KCNQ2* was decreased significantly following PC12 cells transfection with miR-27a-3p mimics.

**Conclusion:** The hub ion channel-related DEGs in mTLE and the miRNA-mRNA regulatory networks had been identified. Moreover, the network of miR-27a-3p regulating ion channel genes will be of great value in mTLE.

## Introduction

There were currently about 65 million patients affected by epilepsy worldwide. More than 80% of the patients with epilepsy occurred in low- and middle-income countries, about 15 per 1,000 people (GBD 2016 Epilepsy Collaborators, 2019). Temporal lobe epilepsy (TLE) was the most common type of adult epilepsy, and its prevalence rate was about 1.7 in 1000 (Englot et al., 2020). Although most TLE patients were treated with regular medication, about 40% of seizures cannot be controlled, which significantly decreased the quality of life for patients and placed a heavy burden on society (Englot et al., 2020). The pathogenesis of epilepsy was very complex. With the improvement of genetic screening techniques, the construction of large-scale genetic databases, and the popularization of gene sequencing, multiple genes closely related to brain network remodeling, neuronal death, inflammation, changes in the function of ionic channels and neurogenesis were identified for epileptogenesis (Henshall et al., 2016).

A systematic review summarized 977 epilepsy-related genes, in which ion channels and receptors occupied the major part (Wang et al., 2017). Furthermore, disruption of ion channels could cause a wide spectrum of human diseases, known as channelopathies (Palleria et al., 2015). Ion channels were not only the basis for generating and regulating the excitability of neurons but also involved in maintaining cellular ion homeostasis and membrane potential. Various ion channels associated with epilepsy had been identified, including voltage-gated ion channels, ligand-gated ion channels and other ion channels. Some of them were the molecular targets of antiepileptic drugs.

MicroRNA (miRNA) is one of the non-coding RNAs that are capable of regulating gene expression by affecting mRNA stability and inhibiting translation. Its role in epilepsy was becoming more prominent due to its ability to regulate multiple mRNAs simultaneously. In addition, miRNA may not only be involved in the pathological processes of epilepsy but also be considered a potential new therapeutic target to override drug resistance (Brennan and Henshall, 2018; Gross and Tiwari, 2018; Almeida Silva et al., 2020). Prior studies had also reported that miRNAs could alter the excitability of the neuron and effectively influence the incidence of epilepsy (Henshall et al., 2016). Over 1,000 miRNAs expression alterations in the hippocampus and other epileptic focus had been described in human epilepsy and models of epilepsy (Mooney et al., 2016). Manipulation of miRNAs may have powerful effects on seizures and epilepsy-induced neuronal cell death (Dogini et al., 2013). MiRNAs may affect epileptogenesis by altering the silencing and translation of ion channels.

In recent years, research on the molecular regulation mechanism of miRNA in mTLE-related genes had made tremendous progress. However, the details of the mechanism of ion channel-related genes that are mediated by miRNA are worthy of further analysis and investigation. Therefore, this study initially identified the key miRNAs and mRNAs and construct the miRNA-mRNA regulatory network related to ion channels genes in mTLE.

## Materials and Methods

### Data Sources

**FIGURE 1:**
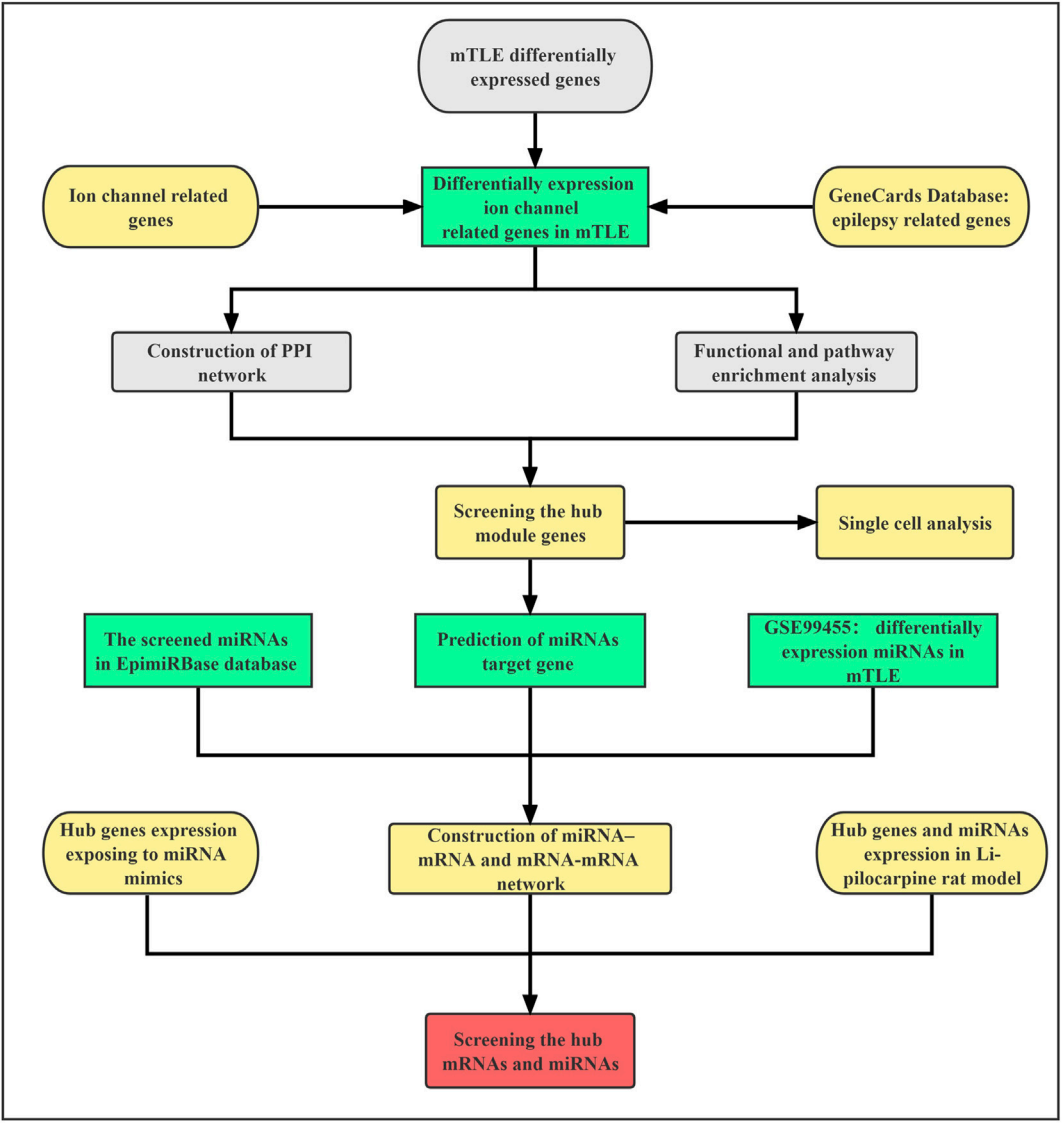
The flow chart.

The workflow of our study was shown in Figure 1. To identify ion channel-related genes associated with mTLE, including ligand-gated ion channel genes, voltage-gated ion channels genes and other ion channel genes, the IUPHAR/BPS Guide to Pharmacology (GTP) database (https://www.guidetopharmacology.org/) and neXtProt database (https://www.nextprot.org/) were searched. Then, the medical subject headings (MeSH) of epilepsy and its text words related to epilepsy were searched in GeneCards database: The Human Gene Database (https://www.genecards.org/), a comprehensive resource for gene-related information, which contains a total of gene-centric data from 150 web sources. Finally, we combined the results with the published high-throughput sequencing results of mTLE in the journal of Brain (Guelfi S et al., 2019) to screen expression profiles of mTLE-related ion genes. The study included temporal lobe cortical tissue samples from 85 patients who were neuropathologically confirmed as definite mTLE + HS and 75 neurologically healthy controls for whole-gene and exon-level transcriptome analysis. The data adjustment was performed using the residual method, and the false discovery rate (FDR) was set to less than 5% (Guelfi et al., 2019).

### DEGs Functional and Signaling Pathway Enrichment Analysis

The mTLE ion channel-related genes were extracted for GO functional enrichment and KEGG pathway enrichment analysis. The analysis method was based on the R clusterProfiler and pathview packages. The results were visually displayed by the online bioinformatics tool.

### Construction of PPI Network and Identification of Hub Module

The PPI networks were produced by STRING (http://string-db.org/). The hub modules were then identified by the cytoHubba plug-in in Cytoscape software. CytoHubba was a topological network algorithm that assigned a value to each gene and sequentially discovered its hub genes and sub-network.

### Single Cell Analysis of Hub Module Genes

Single cell analysis was done using the Single Cell Portal database (https://singlecell.broadinstitute.org/single_cell). The database collected the results of 390 single-cell sequencing studies and contained sequencing data for a total of 17061708 cells. Single-cell raw RNA sequencing data were retrieved under accession of brain tissue. The distribution of hub module genes in normal hippocampal tissue and their expression in different cell types were analyzed.

### Prediction of miRNA-Targeting Hub Module Genes

Visualization and functional annotation of potential ion channels mRNA-miRNA interactions were performed using the miRNet website online tool (https://www.mirnet.ca/). Its miRNA-mRNA target pairs prediction was from the TarBase database, which is based on experimental validation (Chang et al., 2020).

### Screening of Differentially Expressed miRNAs in mTLE

To screen for differentially expressed miRNAs in mTLE, we searched the NCBI Gene Expression Omnibus (GEO) database to retrieve any appropriate dataset associated with epilepsy. The inclusion criteria were 1) the specimens were from brain tissue; 2) the specimens included mTLE patients and normal control; 3) the species were limited to *Homo sapiens*; 4) the raw data or processed data were public and accessible. The GSE99455 dataset was selected as a candidate to study ultimately. The detailed information of GSE99455 was shown in Supplementary Table S1. We processed the Bioconductor software package Deseq2 to identify differentially expressed miRNAs. We selected those miRNAs using selection with |log2FC|> 0.5 and FDR<0.05.

### Identification of the Regulatory Network of miRNA With Hub Module Genes

The miRNAs in the regulatory network were identified from differentially expressed miRNAs in mTLE, prediction result of miRNA targeting hub module genes and the EpimiRBase database. The EpimiRBase database (https://www.epimirbase.eu/epimirbase/), which contains differentially expressed miRNAs associated with epilepsy, including results from experimental studies in *Homo sapiens*, rats and mice. The miRNA with the most regulatory targets were selected for the next step of the study.

### Establishment of the Lithium-Pilocarpine Chronic Epilepsy Model

Sixteen healthy male Sprague-Dawley (SD) rats, aged 8–9 weeks and weighing 150–180 g, were purchased from Beijing Vital Lihua Experimental Animals Co., Ltd.

All animals were raised in SPF level environment (circadian rhythm: 12 h/12 h, temperature: 22 ± 2°C; humidity: 50 ± 10%) and were allowed free access to food and water. Prior to initiation of experimental procedures, rats were acclimatized for at least a 3-day period. All procedures were in accordance with the Regulations of Experimental Animal Administration issued by the Ministry of Science and Technology of the People’s Republic of China (http://www.most.gov.cn). The rats were injected intraperitoneally with lithium chloride (127 mg/kg, Sigma L9650) on day 1 and administered scopolamine methyl bromide (1 mg/kg, Macklin S835305) injection 24 h later, Intraperitoneal injected of pilocarpine (TargetMol T0804) 30 min later. The first dose of pilocarpine was 30 mg/kg intraperitoneally. If there was no grade IV or above seizure grade after 30 min, an additional dose of 10 mg/kg would be given intraperitoneally at 30 min interval until there was grade IV or above Status epileptics (SE)-like seizure without obvious interval, and the extreme dose was 60 mg/kg. SE was terminated after 2 h by injecting diazepam (10 mg/kg). The rat seizure symptoms were graded based on the Racine grading criteria. Animals were video-monitored 8 h a day for general behavior and occurrence of spontaneous seizures by 2 weeks after SE. Rats showing spontaneous recurrent seizures were used as chronic epilepsy animals, which were randomly chosen for EEG recording.

### Cell Culture and Transfection

PC12 cells were ordered from Zixiao Biological Technology Co., Ltd. (Wuxi, China). The above cells were grown in RPMI-1640 complete medium (Thermo Fisher Scientific, Shanghai, China) comprising 10% fetal bovine serum and 1% penicillin/streptomycin. They were then maintained at 37°C with 5% CO_2_ and saturated humidity, with the medium substituted once every 2–3 days. During the cells’ logarithmic growth phase, 0.25% trypsin (Thermo Fisher HyClone, Utah, United States) was adopted for trypsinization and passage. Afterward, the cells were trypsinized with 0.25% trypsin for subsequent experiments. The above cell lines were inoculated into 6-well plates and further cultured in an incubator. When the cells reached more than 80% confluence, they were transfected with miR-27a-3p mimics and miR-27a-3p negative controls, followed by 24 h of culturing. After trypsinization with 0.25% trypsin, the cells were used for subsequent experiments Real-time fluorescent quantitative PCR (RT-qPCR).

### RT-qPCR

The hippocampus of brain tissue was removed after rapid decapitation of SD rats, and the tissue was isolated on ice. Total RNA was extracted using the miRcute miRNA Isolation Kit (DP501, Tiangen, China) and subsequently reversed transcribed using the miRcute Plus miRNA First-Strand cDNA Kit (KR211, Tiangen, China) and FastKing gDNA Dispelling RT SuperMix Kit (KR118, Tiangen, China). RT-qPCR was performed on the ABI QuantStudio5 System using miRcute Plus miRNA qPCR Kit (SYBR Green) (FP411, Tiangen, China) and Talent qPCR PreMix Kit (SYBR Green) (FP209, Tiangen, China). The primer design was completed by Sangon Biotech, and the primer list was shown in Supplementary Table S2.

### Statistical Analysis

Most of the statistical results were completed through R software, log2FC > 0.5 and *p*-value <0.05 were considered statistically significant when performing gene expression differential analysis. GraphPad Prism 9 software was used for the *t*-test and the visual display was mainly based on Cytoscape software.

## Results

### Genes Associated With Ion Channels in mTLE

**FIGURE 2:**
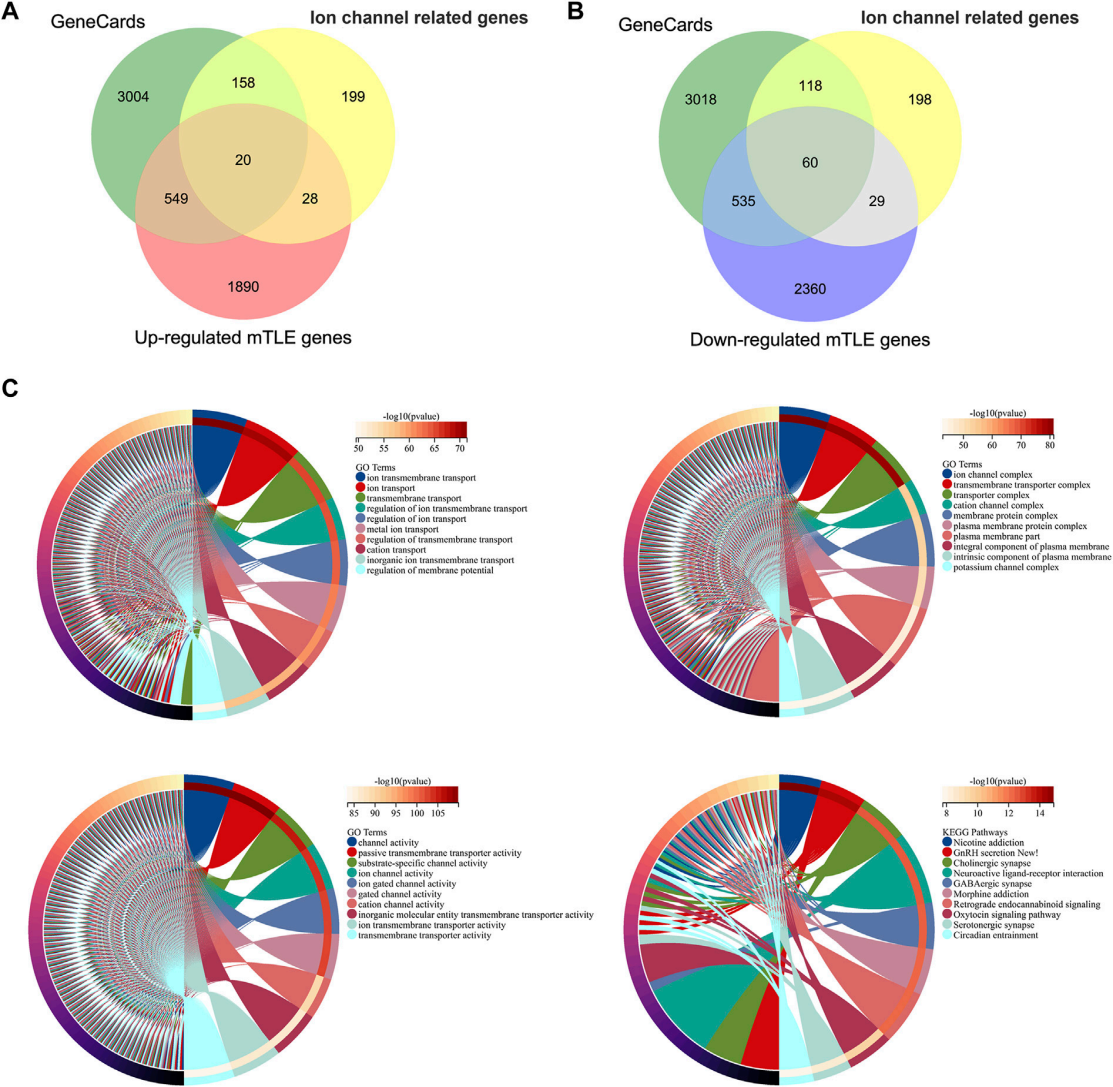
Ion channel-related genes of mTLE and functional enrichment analysis. **(A,B)** Venn diagrams of the screened ion channel-related DEGs in mTLE. The green part indicated the epilepsy-related genes screened in the GeneCards database, the yellow part indicated the ion channel-related genes, and the red and blue parts indicated the dysregulated genes with up and down expression in mTLE. **(C)** Functional enrichment analysis of mTLE ion channel-related genes. The chord diagram showed the Top10 analysis enrichment results.

A total of 405 ion channel-related genes were taken into account. These genes were classified into three types: ligand-gated ion channels, voltage-gated ion channels, and other ion channels. 3732 genes related to epilepsy were screened in the GeneCards database. Subsequently, a total of 80 differentially expressed mTLE-related ion genes were screened out with 20 high-expressed genes and 60 low-expressed genes. The results were shown in Figures 2A,B. For details, see Supplementary Table S3.

### Functional and Pathway Enrichment Analysis

80 DEGs related to mTLE were analyzed further by GO term and KEGG pathway analysis. The biological processes were concentrated in regulating membrane potential, regulation of ion transmembrane transport, and potassium ion transmembrane transport. The analysis result of the cellular component focused on ion channel complex, transmembrane transporter complex, transporter complex, and cation channel complex. Passive transmembrane transporter activity and ion channel activity were mainly enriched in molecular function. The result of the KEEG pathway analysis focused on neuroactive ligand-receptor interaction, GnRH secretion, GABAergic synapse and oxytocin signaling pathways. The functional enrichment analysis results of these genes mainly focused on a variety of voltage-gated channel activation, ion transport across the membrane, and other critical biological processes (Figure 2C).

### Hub Module of mTLE Ion Channel-Related Genes

**FIGURE 3:**
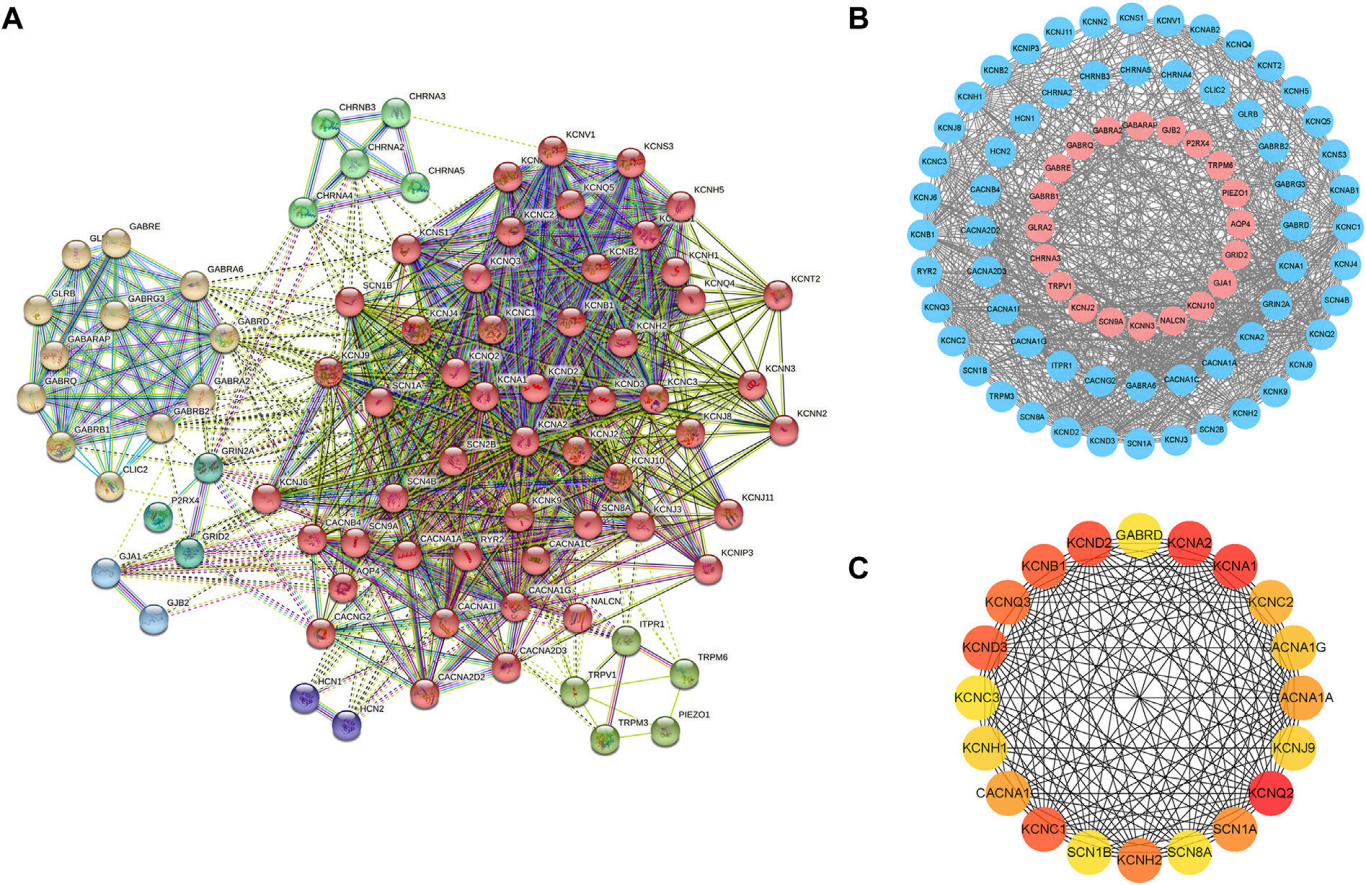
PPI network of mTLE ion channel-related genes and hub module genes **(A)** The entire mTLE ion channel-related genes were divided into four main modules. **(B)** PPI network of mTLE ion channel-related genes, red dots represent the up-regulated mTLE ion channel-related genes, blue represents down-regulated mTLE ion channel-related genes, and black lines show the existence of interaction between those encoding proteins. **(C)** The hub module genes PPI network.

The PPI network was constructed based on the STRING database for ion channel-related genes in mTLE, which were broken down into four main modules (Figures 3A,B). The hub module (20 genes) had been screened in the PPI network (Figure 3C), which had higher linkage in the whole PPI network and might play a more important role in physiological processes. Information about the hub module genes were shown in Table 1.

**TABLE 1:** The hub module genes.

Genes	Type	*p* value	LogFC	Expression
*CACNA1A*	vgic	0.0004	−1.1127	Down
*CACNA1C*	vgic	0.0168	−1.0732	Down
*CACNA1G*	vgic	0.0001	−1.1708	Down
*GABRD*	lgic	0.0000	−1.2457	Down
*KCNA1*	vgic	0.0000	−1.4105	Down
*KCNA2*	vgic	0.0000	−1.2605	Down
*KCNB1*	vgic	0.0000	−1.2777	Down
*KCNC1*	vgic	0.0001	−1.1897	Down
*KCNC2*	vgic	0.0101	−1.1167	Down
*KCNC3*	vgic	0.0000	−1.1720	Down
*KCND2*	vgic	0.0001	−1.1699	Down
*KCND3*	vgic	0.0082	−1.0765	Down
*KCNH1*	vgic	0.0001	−1.1568	Down
*KCNH2*	vgic	0.0003	−1.0774	Down
*KCNJ9*	vgic	0.0000	−1.1927	Down
*KCNQ2*	vgic	0.0017	−1.0869	Down
*KCNQ3*	vgic	0.0007	−1.0994	Down
*SCN1A*	vgic	0.0000	−1.4172	Down
*SCN1B*	vgic	0.0000	−1.5477	Down
*SCN8A*	vgic	0.0002	−1.1585	Down

### Single Cell Analysis of Hub Module Genes

**FIGURE 4:**
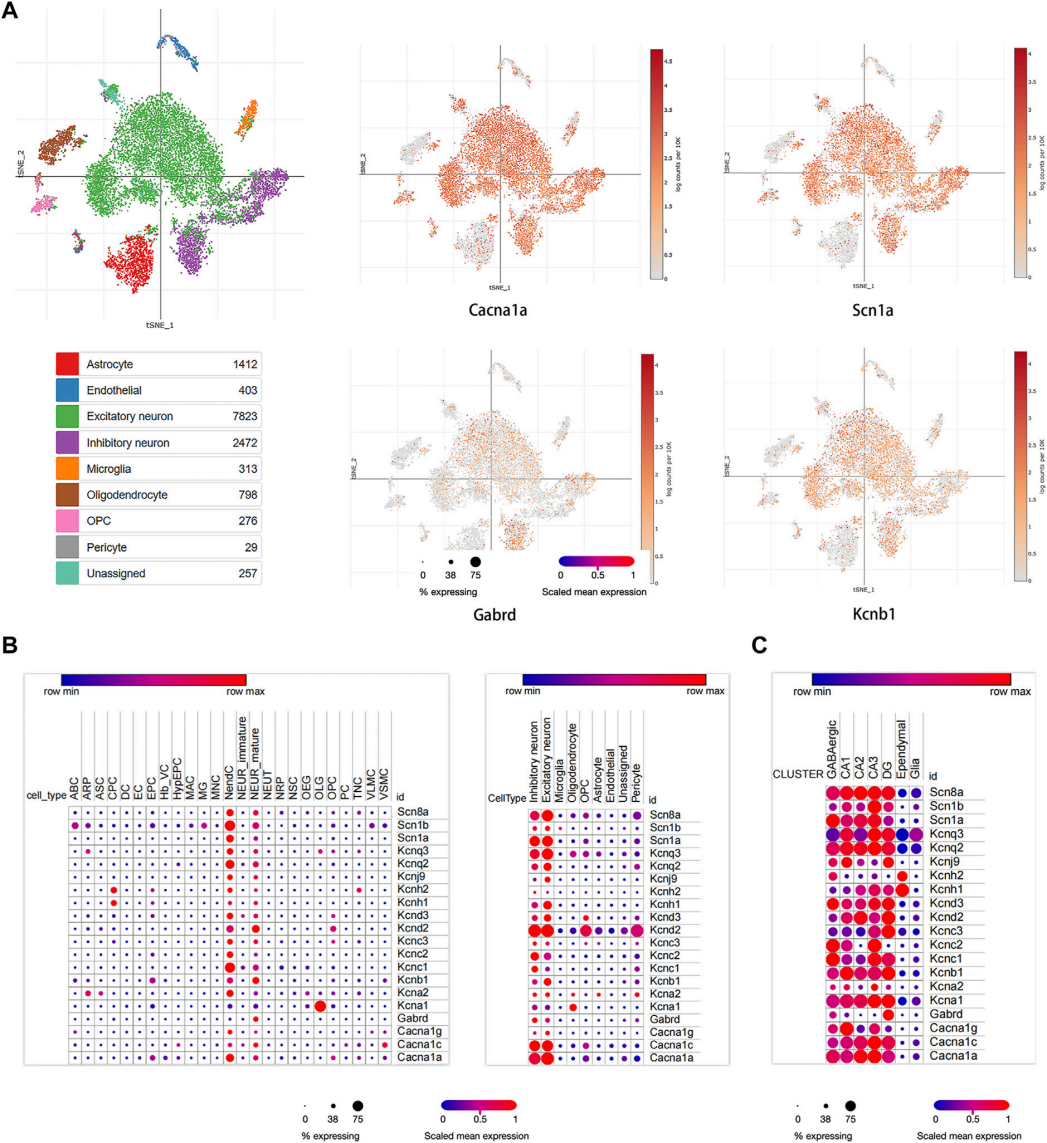
Single cell analysis results of hub module genes. **(A)** The proportion of diverse cell types across hub module genes in the brain; **(B)** the distribution of some ion channel genes in different cell types. **(C)** The expression of hub module genes in different areas of the hippocampus.

Three studies were used to perform single-cell RNA-seq analysis (Habib et al., 2016; Ximerakis et al., 2019; Ding et al., 2020). In our study, we identified nine main clusters (Figure 4A). Based on the expression of ion channel markers, which were strongly and specifically marked regarding each major cell population, we noticed that most of the hub module genes were highly expressed in excitatory neurons, such as *KCNQ3, KCNQ2, KCND3, KCNA2, CACNA1C, KCNJ9, KCND2, KCNB1, CACNA1G*; Genes of *KCNC1, SCN1A, KCNH2, CACNA1C, KCNC2, KCNC3,* and *GABRD* were relatively abundantly expressed in inhibitory neurons; *KCNH1* and *KCNH2* were mainly expressed in cardiac progenitor cells (CPC), and *KCNA1* was mainly located in oligodendrocytes (OLG) (Figures 4A,B). Moreover, we found that the distribution of most hub module genes in the hippocampus, such as *SCN8A, SCN1B, SCN1A, KCNQ3, KCNQ2, KCNH2, KCNH1, KCND3, KCNC2, KCNC1, KCNA1, KCNA2, CACNA1C, CACNA1A*, were enriched in CA3 region of the hippocampus; The expression of *KCNJ9, KCND2, KCNC3, KCNB1, GABRD* were relatively high in DG region; *KCNB1, CACNA1G* were mainly enriched in CA1 region (Figure 4C). Those revealed that the hub genes were mainly located in the CA3 region.

### Differentially Expressed miRNAs and miRNAs Targeting of the Hub Module Genes

**FIGURE 5:**
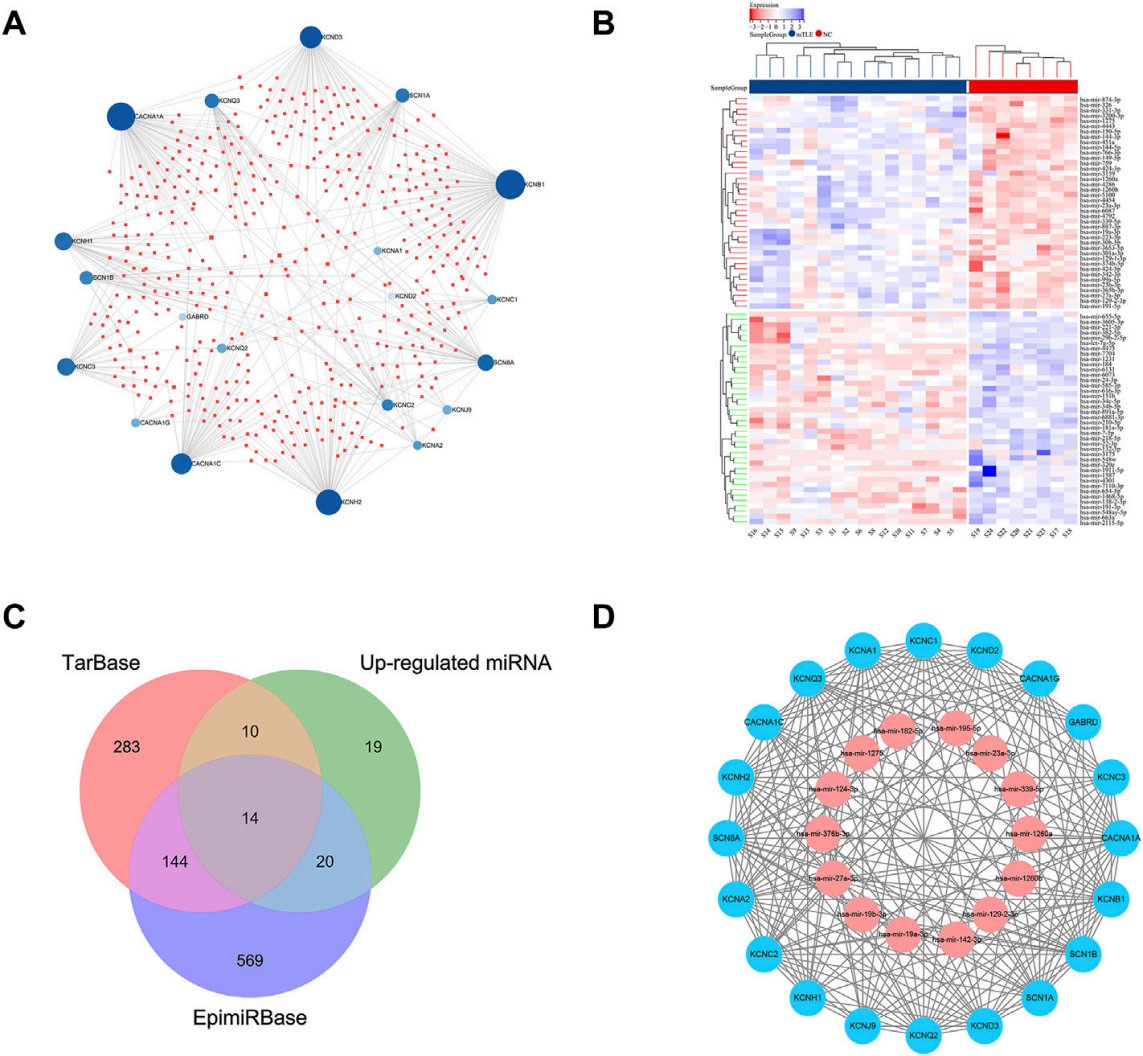
miRNA-mRNA regulatory network of hub module genes. **(A)** Predicted results of target-binding miRNAs based on the TarBase database for the screened hub module genes. Blue dots represented hub module genes in mTLE, red squares represented miRNAs with regulatory relationships. **(B)** The heat map of dataset GSE99455 differentially expressed miRNAs. The horizontal axis showed the sample information (mTLE group and the NC group), and the vertical axis showed the differentially expressed miRNAs. The red color represented the up-regulated miRNAs, and the blue color represented the down-regulated miRNAs. **(C)** Venn diagram of up-regulated expression miRNAs in dataset GSE99455, TarBase prediction results and EpimiRBase database. **(D)** The screened mTLE hub module genes and their target-binding miRNAs; the red color represented the up-regulated genes and the blue color represented the down-regulated genes.

The prediction of miRNAs targeting the hub module genes based on the TarBase database. 451 miRNAs with possible regulatory relationships of hub module genes were predicted (Figure 5A). A total of 103 differentially expressed miRNAs were found in dataset GSE99455 (63 upregulated and 40 down regulated miRNAs) (Figure 5B). Differentially expressed miRNAs information was shown in Supplementary Table S4.

### miRNA-mRNA Regulatory Network

14 miRNAs with a significant regulatory relationship with hub module genes were found, which were proved to be up-regulated expression in mTLE in humans and animal models, the results shown in Figure 5C; Table 2. Figure 5D showed the visualization of the miRNA-mRNA regulatory network which contained 14 miRNAs and 20 mRNAs.

**TABLE 2:** Hub module genes-targeting miRNAs.

miRNA	logFC	*p* value	FDR	Exprssion
hsa-miR-1275	2.7125	0.0000	0.0000	Up
hsa-miR-376b-3p	1.9484	0.0006	0.0100	Up
hsa-miR-23a-3p	0.7900	0.0000	0.0007	Up
hsa-miR-142-3p	1.8311	0.0000	0.0008	Up
hsa-miR-129-2-3p	1.7577	0.0000	0.0000	Up
hsa-miR-182-5p	1.5401	0.0010	0.0128	Up
hsa-miR-19b-3p	1.4601	0.0001	0.0028	Up
hsa-miR-1260b	1.4220	0.0000	0.0000	Up
hsa-miR-19a-3p	1.3845	0.0002	0.0036	Up
hsa-miR-1260a	1.2250	0.0000	0.0000	Up
hsa-miR-27a-3p	0.5830	0.0017	0.0194	Up
hsa-miR-124-3p	0.5721	0.0041	0.0406	Up
hsa-miR-195-5p	1.1019	0.0003	0.0052	Up
hsa-miR-339-5p	1.0530	0.0000	0.0000	Up

### The Network and Expression of miR-27a-3p With Targeted mRNA

**FIGURE 6:**
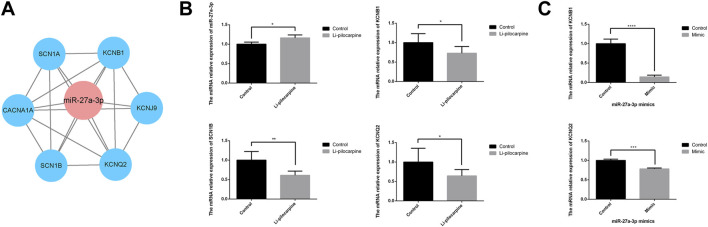
The network and expression of miR-27a-3p with targeted mRNA. **(A)** The miR-27a-3p with targeted mRNA regulatory network. **(B)** Relative expression of miR-27a-3p and mRNA in the hippocampus of lithium-pilocarpine rat epilepsy model. **(C)** Relative expression of *KCNB1* and *KCNQ2* in the PC12 cells after treating with miR-27a-3p mimics. *p value < 0.05, **p value < 0.01, ****p value < 0.0001.

The prediction regulated network of miR-27a-3p and mRNAs showed in Figure 6A. In the study, 62.5% (10/16) of rats displayed spontaneous seizures 60 days after SE. According to the Racine grading standard, epileptic seizures in rats reached grade III -V with obvious epileptic discharge in the hippocampus. The mortality rate was 25% (4/16). Then, it showed that the expression level of miR-27a-3p was increased and the mRNAs expression level of *KCNB1*, *SCN1B* and *KCNQ2* were decreased in lithium–pilocarpine-induced epilepsy models compared with that in the control group (Figure 6B). The mRNAs expression level of *KCNB1* and *KCNQ2* were decreased using RT-qPCR following PC12 cells transfection with miR-27a-3p mimics (Figure 6C).

## Discussion

A growing body of research suggested that large-scale alterations in the expression of genes that controlled ion channels, dendritic remodeling, neuroinflammation and neuronal death were involved in the pathogenesis of epilepsy (Thom, 2014; Srivastava et al., 2017; Bruxel et al., 2021). Ion channels were the basis of excitability regulation, and many human idiopathic epilepsies were currently considered to be ion channel-related disorders (Oyrer et al., 2018). With the gradual deepening of gene research, the role of regulatory networks involving multiple genes in disease has received increasing attention. In the study of temporal lobe epilepsy, genes related to ion channels, inflammation, gliosis, and synaptic structure were involved in the process of seizure and recurrence, and such alterations in differential gene expression affect the whole network (Johnson et al., 2015). The different ion channels were interconnected in time and space to work together. For example, Na^+^ channels worked dynamically in concert with K^+^ channels to generate action potentials (Smith and Walsh, 2020). Therefore, when studying the role of ion channels in temporal lobe epilepsy, we should not only focus on individual channel or a single gene but also the role of multiple channels or multiple genes in epilepsy together as a network are noteworthy and a concern. In this study, we identified 80 ion channel-related genes in mTLE, including 20 high-expressed genes and 60 low-expressed genes, which represented the possible interrelationship among those genes, and performed systematically investigating the contribution of ion channels in the development and formation of epilepsy.

MiRNA was a small non-coding, single-stranded small RNA, which was found in most eukaryotes and was an important mode of epigenetic modification. After processing, mature miRNAs mainly enter the complex by binding to the RNA-induced silencing complex (RISC), which in turn bind specifically to the 3′UTR region of the target gene mRNA in full or partial complementary pairing, thereby enabling regulation of the target gene expression, thus exerting their gene silencing function and ultimately leading to a reduction in the expression of the gene (Lu and Rothenberg, 2018). The miRNA can negatively regulate multiple target genes, and the same target gene can be regulated by multiple miRNAs. It was because miRNAs acted by regulating the expression of target genes, and miRNAs mediate interactions between genes and form complex miRNA-mRNA regulatory networks. Through the study of miRNAs, we can better reveal the important mechanisms of epileptogenesis and find a possible diagnostic marker for early stages and new safe and effective therapeutic targets for epilepsy. In this research, we identified 103 differentially expressed miRNAs with 63 up-regulation and 40 down-regulation in mTLE. miR-27a-3p deserve some attention. This miRNA was shown to regulate multiple ion channels genes. Raoof et al. suggested that miR-27a-3p may be potential diagnostic biomarkers for TLE, and the findings were further confirmed in animal models of epilepsy (Raoof et al., 2018). They found that inhibition of miR-27a-3p could inhibit hippocampal neuronal apoptosis, promote *Bcl2* expression, and decrease *Bax* and *Caspase 3* expression in the kainic acid-induced rat model of epilepsy, meanwhile effectively reducing the expression levels of interleukin-1β (*IL-1β*), *IL-6*, and tumor necrosis factor-α (*TNF-α*) in hippocampal tissues. Previous preliminary experiment data and other research results showed that miR-27a-3p inhibitors prevented epilepsy-induced inflammatory responses and hippocampal neuronal apoptosis by targeting *MAP2K4* (Lu et al., 2019).

In recent years, an increasing number of studies have shown that miRNA played an important role in regulating ion channel silencing. MiRNAs regulated the intrinsic excitability of neurons by targeting ion channels, thereby affecting the entire brain network. A microRNA that regulated voltage-gated potassium channels in the brain was first reported by Raab-Graham et al. They found that miR-129-5p inhibited the expression of the shaker-like potassium channel *Kv1.1* (Sosanya et al., 2013). In the subsequent studies, miRNA-regulated ion channels attracted much attention (Zhang et al., 2018; Tiwari et al., 2019). In our study, we found that the expression level of miR-27a-3p was increased and the mRNAs expression level of *KCNB1, SCN1B* and *KCNQ2* were decreased significantly in epilepsy models. In addition, miR-27a-3p could down-regulate mRNAs expression levels of *KCNB1* and *KCNQ2*, which showed that miR-27a-3p could regulate multiple ion channel genes. Moreover, Single cell analysis results also revealed that *KCNB1* and *KCNQ2* were highly expressed in excitatory neurons. Potassium channels were the largest family of ion channel proteins that regulated changes in cell membrane currents. Their main function was to maintain the resting potential of the cell membrane and to control cell excitability by mediating the repolarization of the cell membrane through the outflow of potassium ions. The *KCNB1* gene encoded the *Kv2.1* channel protein, a major component of the somatodendritic delayed rectifier-type potassium channel in hippocampal and cortical neurons, with a high density of distribution in hippocampal neurons. Its physiological functions included mediating the efflux of potassium currents, being essential for apoptotic signaling cascades as well as maintaining membrane potential, and regulating the electrical excitability of neurons and muscles (Zhang et al., 2018). Torkamani et al. identified three different *de novo* heterozygous missense mutations in the *KCNB1* gene in three unrelated patients with developmental and epileptic encephalopathies. All mutations affected the pore domain of the channel, and *in vitro* expression studies in CHO-K1 cells had shown that these mutations result in a loss of potassium ion selectivity while gaining depolarized cation inward conductivity (Torkamani et al., 2014). In animal models, *KCNB1* knockout mice were found to be more excitable than normal mice, as well as to promote the development of epilepsy, thus reinforcing the important role of *KCNB1* in epileptogenesis (Speca et al., 2014). The *KCNQ2* gene encodes the *Kv7.2* protein, together with its homologue *KCNQ3,* forms homo- and heterotetrameric ion channels in the neuronal plasma membrane, and is responsible for the M-current, thereby preventing neuronal hyperexcitability (Li et al., 2021). Abnormalities of *KCNQ2* ion channel proteins may lead to benign familial neonatal epileps (Singh et al., 1998). Abnormalities in this channel could also cause epileptic encephalopathy. Many patients usually present with recurrent seizures. They have the characteristics of long seizure-free periods, and followed by seizure recurrences, along with severe intellectual impairment and language dysfunctions (Boets et al., 2021). *KCNQ2* was also a very crucial target for antiepileptic drug research, and retigabine was an antiepileptic drug developed specifically for the *KCNQ* channel, but its treatment was sub-optimal, and miRNA may be a better therapeutic option.

Previous studies on the regulation of ion channel gene expression by miRNAs had focused on the effect of a particular miRNA on one ion channel gene, but there was growing evidence to support the involvement of numerous ion channel genes in the pathogenesis of epilepsy. In this study, to further investigate the regulatory network of ion channel genes and miRNAs in mTLE, we identified miRNAs that can regulate multiple important ion channels. Therefore, establishing the network of miRNAs regulating ion channels is of great significance in mTLE. In this study, we predicted the related network regulatory relationships by clustering the alterations of ion channel genes in the important pathological process of epilepsy pathogenesis, which is an important insight for the future exploration of other related pathological processes in epilepsy regarding the network regulation of miRNAs in their related expression genes, such as ion channel alterations, neuroinflammation and neuronal loss et al.

The miRNAs and their regulatory network study will have profound implications at the step of the drug development process for anti-seizures. Meanwhile, our study provides new insights into the understanding of miRNA-mediated alterations that may be critical for the onset and progression of epilepsy.

## Conclusion

The hub mTLE ion channel-related genes and miRNAs that may regulate multiple important ion channels had been identified, while the network of miR-27a-3p regulating potassium ion channel genes was established in mTLE. Based on the miRNA-mRNA regulatory network, it is suggested that the above-mentioned ion channel genes have an important role in the pathogenesis of mTLE and the miRNA has a potential targeting role in its regulation.

### Limitations

Although our study explores ion channel genes and their regulatory networks at the cellular and animal levels, it lacks phenotypic studies in epilepsy patients. In addition, how the above miRNAs are involved in the occurrence of ion channel gene expression downregulation in mTLE and the mechanisms of their involvement in regulation have not been elucidated, so it is worthwhile to investigate and explore in-depth the regulation of ion channel gene expression by miRNAs in mTLE.

## Data Availability

The datasets presented in this study can be found in online repositories. The names of the repository/repositories and accession number(s) can be found in the article/Supplementary Material.
