# Supplementary material for: Identification of Ion Channel-Related Genes and miRNA-mRNA Networks in Mesial Temporal Lobe Epilepsy
Source: Front Genet. 2022 Mar 29;13:853529. doi: 10.3389/fgene.2022.853529 (PMC9001885; doi:10.3389/fgene.2022.853529)
Supplement: Supplementary file 1 [file Table5.DOCX]

Supplement table 3. The information of genes associated with ion channels in temporal lobe epilepsy.

| *Gene Symbol* | p-value(Disease) | Fold-Change(MTLE vs. Control) |
| --- | --- | --- |
| *AQP4* | 7.51E-09 | 1.40083 |
| *CHRNA3* | 0.00920974 | 1.08835 |
| *GABARAP* | 3.35E-05 | 1.06225 |
| *GABRA2* | 0.00741165 | 1.10288 |
| *GABRB1* | 0.000489992 | 1.13205 |
| *GABRE* | 0.0165127 | 1.13234 |
| *GABRQ* | 4.57E-06 | 1.3575 |
| *GJA1* | 5.57E-05 | 1.3064 |
| *GJB2* | 0.0036599 | 1.17743 |
| *GLRA2* | 0.000526841 | 1.20519 |
| *GRID2* | 1.56E-07 | 1.16064 |
| *KCNJ10* | 0.000576183 | 1.16855 |
| *KCNJ2* | 0.00991751 | 1.15772 |
| *KCNN3* | 1.18E-05 | 1.18265 |
| *NALCN* | 0.00184562 | 1.03745 |
| *P2RX4* | 0.000115225 | 1.07151 |
| *PIEZO1* | 0.00310476 | 1.08613 |
| *SCN9A* | 5.24E-05 | 1.14444 |
| *TRPM6* | 0.000781904 | 1.16521 |
| *TRPV1* | 1.82E-05 | 1.19071 |
| *CACNA1A* | 0.0004 | -1.1127 |
| *CACNA1C* | 0.0168 | -1.0732 |
| *CACNA1G* | 0.0001 | -1.1708 |
| *CACNA1I* | 1.09E-05 | -1.14444 |
| *CACNA2D2* | 3.20E-09 | -1.15527 |
| *CACNA2D3* | 2.76E-06 | -1.17243 |
| *CACNB4* | 8.37E-05 | -1.15292 |
| *CACNG2* | 0.000839315 | -1.17776 |
| *CHRNA2* | 4.21E-05 | -1.19716 |
| *CHRNA4* | 0.000340667 | -1.09443 |
| *CHRNA5* | 0.00312821 | -1.13939 |
| *CHRNB3* | 0.00230675 | -1.12672 |
| *CLIC2* | 0.00585357 | -1.07849 |
| *GABRA6* | 0.00459292 | -1.08757 |
| *GABRB2* | 0.000817741 | -1.17442 |
| *GABRD* | 0.0000 | -1.2457 |
| *GABRG3* | 8.03E-05 | -1.18066 |
| *GLRB* | 8.16E-05 | -1.24584 |
| *GRIN2A* | 0.00307028 | -1.13325 |
| *HCN1* | 0.00759419 | -1.17565 |
| *HCN2* | 2.64E-11 | -1.26578 |
| *ITPR1* | 1.59E-08 | -1.29419 |
| *KCNA1* | 0.0000 | -1.4105 |
| *KCNA2* | 0.0000 | -1.2605 |
| *KCNAB1* | 5.63E-06 | -1.13192 |
| *KCNAB2* | 0.00292107 | -1.11827 |
| *KCNB1* | 0.0000 | -1.2777 |
| *KCNB2* | 0.000149686 | -1.16736 |
| *KCNC1* | 0.0001 | -1.1897 |
| *KCNC2* | 0.0101 | -1.1167 |
| *KCNC3* | 0.0000 | -1.1720 |
| *KCND2* | 0.0001 | -1.1699 |
| *KCND3* | 0.0082 | -1.0765 |
| *KCNH1* | 0.0001 | -1.1568 |
| *KCNH2* | 0.0003 | -1.0774 |
| *KCNH5* | 0.000221138 | -1.21486 |
| *KCNIP3* | 2.22E-06 | -1.16347 |
| *KCNJ11* | 2.39E-07 | -1.24058 |
| *KCNJ3* | 0.00109251 | -1.17375 |
| *KCNJ4* | 8.19E-06 | -1.22178 |
| *KCNJ6* | 0.000955268 | -1.13927 |
| *KCNJ8* | 0.00142971 | -1.09467 |
| *KCNJ9* | 0.0000 | -1.1927 |
| *KCNK9* | 0.00385444 | -1.10905 |
| *KCNN2* | 0.00293227 | -1.08096 |
| *KCNQ2* | 0.0017 | -1.0869 |
| *KCNQ3* | 0.0007 | -1.0994 |
| *KCNQ4* | 8.46E-05 | -1.11203 |
| *KCNQ5* | 0.000391306 | -1.20889 |
| *KCNS1* | 5.09E-08 | -1.47137 |
| *KCNS3* | 3.39E-08 | -1.19683 |
| *KCNT2* | 2.13E-08 | -1.25844 |
| *KCNV1* | 9.74E-05 | -1.21875 |
| *RYR2* | 0.0120905 | -1.08648 |
| *SCN1A* | 0.0000 | -1.4172 |
| *SCN1B* | 0.0000 | -1.5477 |
| *SCN2B* | 2.05E-08 | -1.21394 |
| *SCN4B* | 1.79E-08 | -1.27974 |
| *SCN8A* | 0.0002 | -1.1585 |
| *TRPM3* | 0.00425303 | -1.09632 |
