# Supplementary material for: Identification of Ion Channel-Related Genes and miRNA-mRNA Networks in Mesial Temporal Lobe Epilepsy
Source: Front Genet. 2022 Mar 29;13:853529. doi: 10.3389/fgene.2022.853529 (PMC9001885; doi:10.3389/fgene.2022.853529)
Supplement: Supplementary file 2 [file Table3.DOCX]

Supplement table 1. The clinical information of GSE99455 dataset

| Samples | Clinical diagnosis | Age | Gender | Tissue | First seizure at the age of | Cause of death |
| --- | --- | --- | --- | --- | --- | --- |
| 1 | mTLE+HS | 46 | M | hippocampus | 17 | - |
| 2 | mTLE+HS | 47 | W | hippocampus | 14 | - |
| 3 | mTLE+HS | 29 | M | hippocampus | 7 | - |
| 4 | mTLE+HS | 51 | W | hippocampus | 23 | - |
| 5 | mTLE+HS | 48 | W | hippocampus | 3 | - |
| 6 | mTLE+HS | 36 | W | hippocampus | 15 | - |
| 7 | mTLE+HS | 47 | W | hippocampus | 17 | - |
| 8 | mTLE+HS | 40 | M | hippocampus | 19 | - |
| 9 | mTLE+HS | 45 | W | hippocampus | 2 | - |
| 10 | mTLE+HS | 45 | W | hippocampus | 44 | - |
| 11 | mTLE+HS | 34 | W | hippocampus | 12 | - |
| 12 | mTLE+HS | 33 | M | hippocampus | 6 | - |
| 13 | mTLE+HS | 49 | W | hippocampus | 39 | - |
| 14 | mTLE+HS | 36 | M | hippocampus | 6 | - |
| 15 | mTLE+HS | 25 | M | hippocampus | 20 | - |
| 16 | mTLE+HS | 32 | M | hippocampus | 27 | - |
| 17 | NC | 61 | W | hippocampus | - | bronchopnemonia |
| 18 | NC | 62 | M | hippocampus | - | heart failure |
| 19 | NC | 53 | M | hippocampus | - | myocardial infrction |
| 20 | NC | 72 | M | hippocampus | - | heart failure |
| 21 | NC | 72 | M | hippocampus | - | heart failure |
| 22 | NC | 50 | M | hippocampus | - | heart failure |
| 23 | NC | 30 | W | hippocampus | - | heart failure |
| 24 | NC | 45 | M | hippocampus | - | heart failure |
