# Supplementary material for: Identification of Ion Channel-Related Genes and miRNA-mRNA Networks in Mesial Temporal Lobe Epilepsy
Source: Front Genet. 2022 Mar 29;13:853529. doi: 10.3389/fgene.2022.853529 (PMC9001885; doi:10.3389/fgene.2022.853529)
Supplement: Supplementary file 3 [file Table4.DOCX]

| **Gene** | **Primer orientation** | **Primer sequence (5’-3’)** |
| --- | --- | --- |
| KCNB1 | Forward | GAGGGCGAGGAGTTTGACAACAC |
|  | Reverse | CGATGGTGGACAGGACGATGAAC |
| KCNJ9 | Forward | CGAGACCTACCGCTACCTGACC |
|  | Reverse | GCACGAAGAAGAGCAGGCTGAG |
| KCNQ2 | Forward | TTGGTGTCTCATTCTTCGCTCTTCC |
|  | Reverse | AGGATTCCGCCGTTTCTCAAAGTG |
| SCN1A | Forward | GCAGCAACAGCATCAGAACATTCC |
|  | Reverse | CCTCCGATTCCGCCTTTCTTTAGC |
| SCN1B | Forward | AGACCTTCACGGAGTGGACCTTC |
|  | Reverse | GCAGCACCTCATTCTCATAGCGTAG |
| CACNA1A | Forward | CTGACTGGCGAGGATTGGAATGAG |
|  | Reverse | AGGGTGAGGACGATGAAGTAGATGG |
| ACTB | Forward | GCATGGGCCAGAAAGTCTCCTACGC |
|  | Reverse | CAGAAGCACTGGGTGTTCCTCGGGC |

Supplement table 2. Primer sequences for real-time quantitative PCR
