# Supplementary material for: Identification of Ion Channel-Related Genes and miRNA-mRNA Networks in Mesial Temporal Lobe Epilepsy
Source: Front Genet. 2022 Mar 29;13:853529. doi: 10.3389/fgene.2022.853529 (PMC9001885; doi:10.3389/fgene.2022.853529)
Supplement: Supplementary file 4 [file Table6.DOCX]

Supplement table 4. The information of differential expression miRNA data

| miRNA | logFC | PValue | FDR |
| --- | --- | --- | --- |
| hsa-mir-1284 | 4.097946767 | 0.001653695 | 0.019242996 |
| hsa-mir-3125 | 3.57321328 | 0.001004309 | 0.012707673 |
| hsa-mir-190a-3p | 3.452520471 | 0.000982987 | 0.012707673 |
| hsa-mir-4725-3p | 3.333728824 | 0.005503037 | 0.048139897 |
| hsa-mir-1275 | 2.71246058 | 6.89E-26 | 6.17E-23 |
| hsa-mir-4454 | 2.494838808 | 8.51E-11 | 1.09E-08 |
| hsa-mir-4792 | 2.300113994 | 3.04E-06 | 0.000118454 |
| hsa-mir-451a | 2.248600125 | 1.44E-08 | 1.29E-06 |
| hsa-mir-3195 | 2.105192749 | 0.002417143 | 0.02673778 |
| hsa-mir-5100 | 0.927792505 | 1.05E-06 | 4.96E-05 |
| hsa-mir-4443 | 2.053350309 | 1.54E-12 | 3.45E-10 |
| hsa-mir-302a-5p | 2.040038447 | 0.000742119 | 0.010945836 |
| hsa-mir-376b-3p | 1.948439104 | 0.00064705 | 0.009995814 |
| hsa-mir-759 | 1.929895642 | 4.98E-06 | 0.000159306 |
| hsa-mir-374b-5p | 1.867269205 | 9.63E-07 | 4.83E-05 |
| hsa-mir-144-3p | 1.848425041 | 3.22E-06 | 0.000120176 |
| hsa-mir-23a-3p | 0.789959897 | 2.29E-05 | 0.000661209 |
| hsa-mir-142-3p | 1.831090707 | 2.72E-05 | 0.000762879 |
| hsa-mir-6087 | 1.768621121 | 9.70E-07 | 4.83E-05 |
| hsa-mir-144-5p | 1.762376091 | 8.17E-05 | 0.001877586 |
| hsa-mir-342-3p | 0.7700164 | 5.92E-05 | 0.001472689 |
| hsa-mir-218-5p | -0.881375668 | 6.28E-05 | 0.001521629 |
| hsa-mir-210-3p | -0.887669026 | 7.92E-05 | 0.001867737 |
| hsa-mir-129-2-3p | 1.757657862 | 3.36E-15 | 1.00E-12 |
| hsa-mir-23b-3p | 0.511392424 | 0.000112694 | 0.002462784 |
| hsa-mir-7-5p | -0.748487812 | 0.000118086 | 0.002519176 |
| hsa-mir-22-3p | -0.611628661 | 0.000125763 | 0.002620549 |
| hsa-mir-142-5p | 1.591941162 | 0.000138738 | 0.00275578 |
| hsa-mir-182-5p | 1.540070464 | 0.001029848 | 0.012815881 |
| hsa-mir-149-5p | 0.588908101 | 0.000241873 | 0.00442282 |
| hsa-mir-30b-5p | 0.928793357 | 0.000251242 | 0.004502258 |
| hsa-mir-766-3p | 0.793506848 | 0.000321229 | 0.005535029 |
| hsa-mir-382-5p | -0.862483024 | 0.000373626 | 0.006157129 |
| hsa-mir-19b-3p | 1.460068002 | 0.00014148 | 0.00275578 |
| hsa-mir-1260b | 1.42201965 | 1.80E-08 | 1.34E-06 |
| hsa-mir-150-5p | 0.62636841 | 0.000567685 | 0.008923606 |
| hsa-mir-19a-3p | 1.384531119 | 0.000188104 | 0.003585975 |
| hsa-mir-3200-3p | 0.639393087 | 0.000700256 | 0.010634397 |
| hsa-mir-33a-5p | 1.376935088 | 0.000554882 | 0.00887811 |
| hsa-mir-4286 | 1.372457346 | 2.88E-12 | 5.17E-10 |
| hsa-mir-132-5p | -0.673577152 | 0.000747932 | 0.010945836 |
| hsa-mir-191-5p | 0.518311149 | 0.00078533 | 0.011169139 |
| hsa-mir-887-3p | 0.529754636 | 0.000798763 | 0.011182685 |
| hsa-let-7g-5p | -0.777869011 | 0.000846849 | 0.011390777 |
| hsa-mir-204-5p | 0.651495472 | 0.000851766 | 0.011390777 |
| hsa-mir-151b | -0.65178503 | 0.000945523 | 0.012458654 |
| hsa-mir-365b-3p | 1.280641361 | 2.08E-05 | 0.000621584 |
| hsa-mir-656-3p | 1.266874267 | 0.004401985 | 0.04151767 |
| hsa-mir-1246 | 1.264459409 | 3.55E-06 | 0.000122316 |
| hsa-mir-654-5p | -0.894337692 | 0.001188567 | 0.014588442 |
| hsa-mir-34a-5p | -0.884446245 | 0.001403828 | 0.016997704 |
| hsa-mir-326 | 0.53408687 | 0.001493622 | 0.017843806 |
| hsa-mir-99a-5p | 1.232042045 | 1.89E-06 | 8.06E-05 |
| hsa-mir-1260a | 1.225038445 | 5.61E-07 | 3.35E-05 |
| hsa-mir-27a-3p | 0.583023194 | 0.00169101 | 0.01942494 |
| hsa-mir-193b-3p | 0.70827573 | 0.002294332 | 0.026021789 |
| hsa-mir-223-3p | 1.198763729 | 4.15E-05 | 0.001127481 |
| hsa-mir-874-3p | 1.178261529 | 4.05E-06 | 0.00013424 |
| hsa-mir-181a-5p | -0.736882724 | 0.002754523 | 0.029381577 |
| hsa-mir-203a-3p | 0.878578622 | 0.002741065 | 0.029381577 |
| hsa-mir-424-5p | 1.176972398 | 5.69E-05 | 0.001457015 |
| hsa-mir-301a-3p | 1.174299067 | 0.00022809 | 0.004257675 |
| hsa-mir-3653-3p | 0.710979051 | 0.003479927 | 0.036255982 |
| hsa-mir-219a-5p | 0.864398199 | 0.003941955 | 0.040136273 |
| hsa-let-7i-5p | -0.863377729 | 0.004057573 | 0.040395392 |
| hsa-mir-181b-5p | -0.715931162 | 0.004031945 | 0.040395392 |
| hsa-mir-124-3p | 0.572086676 | 0.004124873 | 0.040614138 |
| hsa-mir-664a-3p | 0.711284017 | 0.004185272 | 0.040760913 |
| hsa-mir-3943 | 1.143663305 | 0.002750468 | 0.029381577 |
| hsa-mir-195-5p | 1.101884499 | 0.000298805 | 0.005249593 |
| hsa-mir-99b-3p | -0.66154899 | 0.00462073 | 0.041819943 |
| hsa-mir-95-3p | -0.642517005 | 0.004556834 | 0.041819943 |
| hsa-mir-6716-3p | -0.937956041 | 0.004588235 | 0.041819943 |
| hsa-mir-30e-3p | 0.610027482 | 0.004867449 | 0.043612344 |
| hsa-mir-4508 | 1.078072686 | 0.004293195 | 0.040922367 |
| hsa-mir-29a-3p | -0.53063689 | 0.005587667 | 0.048139897 |
| hsa-mir-490-3p | 0.811916481 | 0.005496434 | 0.048139897 |
| hsa-mir-484 | 0.524363555 | 0.00554728 | 0.048139897 |
| hsa-mir-339-5p | 1.052988859 | 5.51E-07 | 3.35E-05 |
| hsa-mir-3653-5p | 1.042357444 | 0.001641368 | 0.019242996 |
| hsa-mir-29b-2-5p | -1.012142154 | 0.000133784 | 0.002724323 |
| hsa-mir-29b-1-5p | -1.013078065 | 0.003637921 | 0.037466409 |
| hsa-mir-221-3p | -1.041116796 | 9.14E-05 | 0.002047669 |
| hsa-mir-191-3p | -1.044865156 | 0.00100697 | 0.012707673 |
| hsa-mir-1468-5p | -1.232156195 | 7.47E-07 | 4.18E-05 |
| hsa-mir-3605-3p | -1.266752323 | 0.000377949 | 0.006157129 |
| hsa-mir-548ay-5p | -1.303524221 | 0.003200683 | 0.033738963 |
| hsa-mir-4301 | -1.606713023 | 2.50E-07 | 1.72E-05 |
| hsa-mir-6881-3p | -1.681221464 | 0.004250169 | 0.040922367 |
| hsa-mir-7110-3p | -1.904156784 | 3.38E-06 | 0.000121086 |
| hsa-mir-34c-5p | -1.955161371 | 7.64E-06 | 0.000236151 |
| hsa-mir-585-3p | -1.976189043 | 0.000757413 | 0.010945836 |
| hsa-mir-663a | -2.115866643 | 1.39E-06 | 6.22E-05 |
| hsa-mir-1911-5p | -2.145888002 | 0.004608668 | 0.041819943 |
| hsa-mir-34b-5p | -2.235872524 | 4.71E-05 | 0.001241712 |
| hsa-mir-184 | -2.272829159 | 5.24E-09 | 5.87E-07 |
| hsa-mir-6826-5p | -2.353156138 | 0.002350601 | 0.026326734 |
| hsa-mir-6131 | -2.398748446 | 1.70E-08 | 1.34E-06 |
| hsa-mir-6073 | -2.423670747 | 2.00E-06 | 8.13E-05 |
| hsa-mir-548w | -2.947915333 | 0.00083264 | 0.011390777 |
| hsa-mir-320e | -3.123578337 | 9.88E-09 | 9.83E-07 |
| hsa-mir-7704 | -3.200437568 | 8.62E-12 | 1.29E-09 |
| hsa-mir-1231 | -5.22109073 | 1.23E-15 | 5.53E-13 |
